# Requisite Knowledge for the Practice of Dentistry

**Published:** 1878-07

**Authors:** Frank M. Odell


### ARTICLE VI.

### *Requisite Knowledge for the Practice of Dentistry.*

BY FRANK M. ODELL, D. D. S., M. D.

Read before the First District Dental Society, S. N. Y.

One gentleman asks: "Who can talk upon such a subject? What is there that anyone can say upon such a subject, to set the ball in motion? Ten chances to one, it will resolve itself into some remarks upon the way to prepare amalgam."

Another soliloquized: "The whole thing can be told in two words,—Every thing! Every thing is proper knowledge preparatory to the practice of dentistry, and no kind or amount of instruction and experience comes amiss."

According to my view of the matter, the second querist is not far out in his reckoning; but the vagueness of his mode of expression, only serves clearly to show in what a sea of protoplasm we are floating; and in the hope that I may be able to poise the ball aforesaid, and initiate its propulsion in a proper direction, I have collected a few thoughts upon paper.

The subject before us revolves itself into a question (for it is hardly to be supposed that we have assembled to-night to dogmatically *pronounce* what must be regarded as such "requisite knowledge," and to send forth our pronunciamento, with the seal of this Society upon it, in the expectation that all practitioners and Societies will meekly bow their acquiescence:) What is the requisite knowledge, &c.?

Whilst agreeing with the second opinion, as far as it goes, I am still of the opinion that we may be able to get at a more practical answer to our query; and, as is usual with me, I will begin in the middle, and take up the second idea, which presents itself first.

Anatomy, at least of the teeth and their surrounding alveolar processes, must, as a prime necessity, be practically familiar to the dentist; for how else can he decently extract, or excavate a cavity in a tooth ?

A smattering of Pathology, sufficient to diagnose the various conditions in which suffering humanity comes under our notice;

A sufficient acquaintance with the Therapeutics of dentistry to be able to exhibit understandingly the most common remedial agents;

An insight into the most superficial laws of Philosophy and Mechanics, to enable one to perform the various operations in which we are wont to expend our energies;

Sufficient acquaintance with Chemical and Electrical laws, to enable us to study compatibility, and avoid at least proximity of incompatible agencies or materials employed in the mouth.

All this and more, it will readily be conceded, is requisite knowledge, before we can be fitted as the merest apologies for dental practitioners.

But preparation for practice in the higher walks of our profession, requires that the foundation-stones of the structure be laid broad and deep; that the anatomical knowledge extend to the whole structure of man not only, but embrace comparative anatomy likewise; and that it be not satisfied with any superficial knowledge, but include that of the vascular and neural systems; that even here it pause not, but dip deeper and deeper into minute anatomy, until even the powerful aid of the microscope fails (inasmuch as we learn thereby only what *comparative* observation can teach,) and the aid of the study of Embryology has to be invoked, in order that we may know how to assist in building up tissue, as well as in tearing it down.

Our knowledge of Pathology, if extended to the outer verge of what is known in general medicine, can be productive of only good to our patients, whose general physical condition is often indicated very clearly, and sometimes preceded, by indications and conditions readily seen by the observant dental practioner.

In a Therapeutical direction, we have by no means plumbed the depths of possibilities. Who can tell what application, not antagonistic to protoplasmic integrity, to make to sensitive dentine, or to the intensely vibrating odontoblasts at the exposed neck of a tooth, where even the touch of the cheek, tongue, or finger-nail, is so excessively painful?

We are expanded reservoirs of naked facts; and, as yet, can give no solution of a query as to causes.

"Requisite—knowledge—for—the—practice—of—Dentistry." I am almost led to a complete indorsement of the second opinion quoted at the commencement of this article, that it embraces every thing; for, when we examine it from any point of view, Medical, Mechanical, Social, Aesthetic, we find no field which does not yield a harvest for the dental student to reap.

And all these departments of knowledge, if faithfully worked up, and fully represented by appropriate ganglionic-bodies within the compass of any one organism, will still fall short of completing the *requisite knowledge* for the practice of dentistry, unless carefully guided by *good judgment*, and that most uncommon thing, *common sense.*—*Dental Miscellany.*

